# Supplementary material for: Estimating HIV incidence and assessing associated risk factors among adults: Evidence from the 2018–2022 HIV vaccine preparedness cohort in Masaka, Uganda
Source: PLoS One. 2026 May 8;21(5):e0348769. doi: 10.1371/journal.pone.0348769 (PMC13155609; doi:10.1371/journal.pone.0348769)
Supplement: S1 Fig — (DOCX) [file pone.0348769.s001.docx]

Screening Test
DETERMINE

Non-Reactive

Reactive

Report HIV Negative

Confirmatory Test
STAT-PAK

Non-Reactive

Reactive

Report HIV positive

Tie Breaker Test
SD BIOLINE

Non-Reactive

Reactive

**Report as** INCONCLUSIVE
**Re-rest after 14 days**

Report HIV Negative

**Supporting 1 Figure: Serial HIV testing algorithm for persons above 18 months of age**
