# Supplementary material for: Estimating HIV incidence and assessing associated risk factors among adults: Evidence from the 2018–2022 HIV vaccine preparedness cohort in Masaka, Uganda
Source: PLoS One. 2026 May 8;21(5):e0348769. doi: 10.1371/journal.pone.0348769 (PMC13155609; doi:10.1371/journal.pone.0348769)
Supplement: S1 Table — (DOCX) [file pone.0348769.s002.docx]

**Supporting 2_Table:**  **Comparison of baseline characteristics between participants who did not attend any follow-up visit and those who did in a HIV vaccine preparedness study at Masaka, Uganda.**

| **Characteristics** | **All enrolled** | **≥1 follow-up visit** | **No follow-up visits** | **P-value** |
| --- | --- | --- | --- | --- |
|  | **N (%)^¥^** | **n (%)^¥^** | **n (%)^¥^** |  |
| **Total** | **1422 (100)** | **1115 (78)** | **307 (22)** |  |
| **Gender** |  |  |  | P<0.001 |
| Male | 368 (26) | 346 (31) | 22 (7) |  |
| Female | 1054 (74) | 769 (69) | 285 (93) |  |
| **Age (years)** |  |  |  | 0.251 |
| ≤24 | 791 (56) | 612 (55) | 179 (58) |  |
| >24 | 631 (44) | 503 (45) | 128 (42) |  |
| **Education** |  |  |  | 0.951 |
| ≤Primary | 862 (61) | 677 (61) | 185 (60) |  |
| ≥Secondary | 560 (39) | 438 (39) | 122 (40) |  |
| **Marital Status** |  |  |  | 0.375 |
| Single | 763 (54) | 595 (53) | 168 (55) |  |
| Married/cohabiting/relationship | 385 (27) | 310 (28) | 75 (24) |  |
| Divorced/ separated/ widowed | 274 (19) | 210 (19) | 64 (21) |  |
| **Religion** |  |  |  | 0.089 |
| Christian | 1068 (75) | 827 (74) | 241 (79) |  |
| Muslim/ other | 354 (25) | 288 (26) | 66 (21) |  |
| **Occupation** |  |  |  | P<0.001 |
| Other^¶^ | 375 (26) | 337 (30) | 38 (12) |  |
| Sex worker | 722 (51) | 509 (46) | 213 (69) |  |
| Salon/Lodge/Bar worker^§§^ | 230 (16) | 181 (16) | 49 (16) |  |
| Subsistence fisheries worker^§§§^ | 95 (7) | 88 (8) | 7 (2) |  |
| **Residence** |  |  |  | P<0.001 |
| Non-fishing village | 1216 (86) | 928 (83) | 288 (94) |  |
| Fishing village | 206 (14) | 187 (17) | 19 (6) |  |
| **Had transactional sex in the last 3 months** |  |  |  | P<0.001 |
| No | 137 (10) | 129 (12) | 8 (3) |  |
| Yes | 1285 (90) | 986 (88) | 299 (97) |  |
| **Number of partners in the last 3 months** |  |  |  | 0.017 |
| ≤5 | 523 (37) | 428 (38) | 95 (31) |  |
| ≥6 | 899 (63) | 687 (62) | 212 (69) |  |

**^¥^**^Column percentages; ¶Other includes (multiple options allowed): Professional/technical worker, sales/service worker, subsistence agricultural worker, craft and related trades worker, house helper/labourer, motorcyclist, unemployed, etc. §§Not sex worker; §§§ Not Sex worker and not Salon/Lodge/Bar worker.^
