# Supplementary material for: Estimating HIV incidence and assessing associated risk factors among adults: Evidence from the 2018–2022 HIV vaccine preparedness cohort in Masaka, Uganda
Source: PLoS One. 2026 May 8;21(5):e0348769. doi: 10.1371/journal.pone.0348769 (PMC13155609; doi:10.1371/journal.pone.0348769)
Supplement: S2 Table — (DOCX) [file pone.0348769.s003.docx]

**Supporting 3_Table: HIV incidence by participant occupation among participants classified in those categorised the “Other” occupational category in Table 1.**

| **Occupation*** | **Frequency (%) (N=337)** | **HIV incident cases**^‡^ | **HIV incidence per 100 person-years** |
| --- | --- | --- | --- |
| Professional/technical worker | 64 (19) | 0 | 0.0 |
| Sales/service worker | 80 (24) | 1 | 1.3 (0.2- 9.4) |
| Subsistence agricultural workers | 55 (16) | 0 | 0.0 |
| Craft and related trades workers | 49 (15) | 0 | 0.0 |
| House helper/labourer | 53 (16) | 1 | 2.1 (0.3 – 14.9) |
| Motorcyclist | 26 (8) | 0 | 0.0 |
| unemployed | 24 (7) | 0 | 0.0 |
| Other occupations, not listed above** | 18 (5) | 1 | 3.8 (0.0 – 26.7) |

*Participants were able to report more than one occupation; hence, the total number of responses exceed 337, the number of study participants in this category; ^‡^Each of the three HIV incident cases is unique and counted only in one occupational category; **one sero-conversion occurred in an office clerk, an occupation reported by <1% of participants categorised as “other”.
