# Supplementary material for: Estimating HIV incidence and assessing associated risk factors among adults: Evidence from the 2018–2022 HIV vaccine preparedness cohort in Masaka, Uganda
Source: PLoS One. 2026 May 8;21(5):e0348769. doi: 10.1371/journal.pone.0348769 (PMC13155609; doi:10.1371/journal.pone.0348769)
Supplement: S3 Table — (DOCX) [file pone.0348769.s004.docx]

**Supporting 4_Table: Risk indicators of HIV acquisition among adults in a HIV vaccine preparedness study, Masaka, Uganda, accounting for repeated risk assessments over time.**

| **Risk indicators**** | | | **HIV incidence** | | | **Univariable analysis** | | **Multi-variable analysis^ψ^** | |
| --- | --- | --- | --- | --- | --- | --- | --- | --- | --- |
|  |  |  | **n** | **PYO** | **IR/100PYO**  **(95% CI))** | **IRR**  **(95% CI)** | **P-value** | **aIRR**  **(95% CI)** | **P-value** |
| **Overall** | | | **21** | **797.8** | **2.6 (1.7 – 4.0)** |  |  |  |  |
| **Gender** | | |  |  |  |  |  |  |  |
|  | | Male | 5 | 426.5 | 1.2 (0.5 – 2.8) | **Ref** |  | **Ref** |  |
|  | | Female | 16 | 371.3 | 4.3 (2.6 – 7.0) | 3.67 (1.35 – 10.02) | 0.011 | 3.32 (0.87 – 12.65) | 0.079 |
| **Age at visit** | | |  |  |  |  |  |  |  |
|  | ≤24 | | 11 | 327.2 | 3.4 (1.9 – 6.1) | 1.58 (0.67 – 3.72) | 0.294 | 1.41 (0.58 – 3.40) | 0.449 |
|  | >24 | | 10 | 470.6 | 2.1 (1.1 – 3.9) | **Ref** |  | **Ref** |  |
| **Occupation** | | |  |  |  |  |  |  |  |
|  | Other§ | | 3 | 339.0 | 0.9 (0.3 – 2.7) | **Ref** |  | **Ref** |  |
|  | Sex worker | | 4 | 161.4 | 2.5 (0.9 – 6.6) | 2.80 (0.63 – 12.51) |  | 1.01 (0.19 – 5.38) |  |
|  | Salon/Lodge/Bar worker | | 10 | 154.6 | 6.5 (3.5 – 12.0) | 7.30 (2.01 – 26.53) |  | 2.98 (0.71 – 12.46) |  |
|  | Subsistence fisheries worker | | 4 | 142.8 | 2.8 (1.1 – 7.5) | 3.16 (0.71 – 14.13) | 0.018 | 2.32 (0.44 – 12.27) | 0.212 |
| **Residence** | | |  |  |  |  |  |  |  |
|  | Non-fishing village | | 12 | 528.4 | 2.3 (1.3 – 4.0) | **Ref** |  | **Ref** |  |
|  | Fishing village | | 9 | 269.4 | 3.3 (1.7 – 6.4) | 1.47 (0.62 – 3.49) | 0.382 | 1.85 (0.64 – 5.36) | 0.257 |
| **Used a condom at last sex (past 3 months)** | | |  |  |  |  |  |  |  |
|  | No | | 17 | 582.8 | 2.9 (1.8 – 4.7) | **Ref** |  |  |  |
|  | Yes | | 4 | 215.0 | 1.9 (0.7 – 5.0) | 0.64 (0.21 – 1.89) | 0.418 |  |  |
| **Had transactional sex (past 3 months)** | | |  |  |  |  |  |  |  |
|  | No | | 8 | 340.3 | 2.4 (1.2 – 4.7) | **Ref** |  |  |  |
|  | Yes | | 13 | 457.5 | 2.8 (1.6 – 4.9) | 1.21 (0.50 – 2.91) | 0.674 |  |  |
| **Used recreational drugs (past 3 months)** | | |  |  |  |  |  |  |  |
|  | No | | 17 | 707.2 | 2.4 (1.5 – 3.9) | **Ref** |  |  |  |
|  | Yes | | 4 | 90.6 | 4.4 (1.7 – 11.8) | 1.83 (0.62 – 5.45) | 0.275 |  |  |
| **Sex after consuming alcohol (past 3 months)** | | |  |  |  |  |  |  |  |
|  | No | | 6 | 512.1 | 1.2 (0.5 – 2.6) | **Ref** |  | **Ref** |  |
|  | Yes | | 15 | 285.7 | 5.2 (3.2 – 8.7) | 4.48 (1.73 – 11.54) | 0.002 | 4.16 (1.60 – 10.85) | 0.004 |
| **Number of partners (past 3 months)** | | |  |  |  |  |  |  |  |
|  | ≤5 | | 15 | 566.9 | 2.6 (1.6 – 4.4) | **Ref** |  |  |  |
|  | ≥6 | | 6 | 230.9 | 2.6 (1.2 – 5.8) | 0.98 (0.38 – 2.53) | 0.969 |  |  |
| **STI diagnosis/treatment (past 3 months)** | | |  |  |  |  |  |  |  |
|  | No | | 13 | 653.2 | 2.0 (1.2 – 3.4) | **Ref** |  | **Ref** |  |
|  | Yes | | 8 | 144.6 | 5.5 (2.8 – 1.1) | 2.78 (1.15 – 6.70) | 0.023 | 2.06 (0.84 – 5.06) | 0.116 |
| **Abnormal genital discharge (past 3 months)** | | |  |  |  |  |  |  |  |
|  | No | | 13 | 602.2 | 2.2 (1.3 – 3.7) | Ref |  |  |  |
|  | Yes | | 8 | 195.6 | 4.1 (2.0 – 8.2) | 1.89 (0.79 – 4.57) | 0.155 |  |  |
| **Genital ulcer (past 3 months)** | | |  |  |  |  |  |  |  |
|  | No | | 16 | 696.1 | 2.3 (1.4 – 3.8) | **Ref** |  |  |  |
|  | Yes | | 5 | 101.7 | 4.9 (2.0 – 11.8) | 2.14 (0.78 – 5.83) | 0.138 |  |  |

**Analysis restricted to study visits where both risk indicator data and HIV test results were available; n, number of HIV infections; PYO, person years of observation; IR, incidence rate; IRR, incidence rate ratio; aIRR, adjusted incidence rate ratio, **^ψ^**Multivariable analyses were adjusted for gender, occupation, residence, age at visit, and other predictors retained in the final model.
