# Supplementary material for: Estimating HIV incidence and assessing associated risk factors among adults: Evidence from the 2018–2022 HIV vaccine preparedness cohort in Masaka, Uganda
Source: PLoS One. 2026 May 8;21(5):e0348769. doi: 10.1371/journal.pone.0348769 (PMC13155609; doi:10.1371/journal.pone.0348769)
Supplement: Attachment — Submitted filename: My Comments to Authors 22 June 2025.pdf [file pone.0348769.s007.pdf]

# Reviewing of the revised version of paper titled

## Estimating HIV incidence and assessing associated risk factors among adults : Evidence from the 2018-2022 HIV vaccine preparedness cohort in Masaka, Uganda.

Thank you again for the opportunity to review the revised version of this paper, which address a study with the potential to make a significant contribution to HIV research, particularly within the framework of the "First 95" initiative. I would like to thank the authors for the responses provided. Overall, most of my comments have been integrated, and the quality of the manuscript has improved accordingly. Nonetheless, some comments issues remain (particularly on methodological aspects) that need further attention from the authors to better improve quality of the work. I also strongly recommend that in their response to reviewer comments, the authors systematically reference the specific line numbers of the revised manuscript. This would greatly facilitate the assessment of their revisions and allow a smoother review process. Below are my additional comments following the authors' responses.

**Lines 154 – 160:** Thank you for these clarifications on my previous comment 5 and the additional details added in the manuscript regarding the screening algorithm and quality control of the HIV testing procedures. My concern was related in the fact that the national algorithm, although highly specific and sensitive, relies on rapid diagnostic tests that target antibodies, which may be present at low quantity during the early stages of infection (i.e., during seroconversion). Consequently, there remains a small probability that participants in the acute phase of infection could have been misclassified as HIV-negative at baseline and inadvertently included in the cohort. It would therefore be appropriate to acknowledge this as a potential limitation in the discussion section.

**Lines 180 – 182:** Thank you to the authors for their clarifications following my previous Comment 6. However, I respectfully disagree with the rationale provided, particularly the argument stating:

"The analytical approach used in this paper is a standard method for analyzing incidence data from a single study site. Similar approaches have been applied in other studies (Ahmed, Lutalo et al. 2001, De Schacht, Mabunda et al. 2014, Westreich, Jamal et al. 2014, Gómez-Olivé, Houle et al. 2020). If this were a multi-site study or one where

the same individual was assessed multiple times for an outcome (e.g., repeated blood pressure measurements), incorporating random effects or using a GEE model would be appropriate. However, in our case, each participant had a single outcome (HIV status at the end of follow-up), and the study was conducted at a single site (Masaka). Therefore, there was no intra-individual variability or clear random effect to account for."

This reasoning is not convincing for the following reasons:

1. The fact that the study was conducted at a single site does not in itself justify ignoring intra-individual variability. Even within a single site, a cohort of participants followed over time inevitably induces within-subject correlation across certain characteristics to each individual.
2. Similarly, the fact that each participant had a single binary outcome (HIV status) does not justify ignoring intra-individual variability, as several covariates (such as age, occupation, education level, marital status, and condom use at last sex (past 3 months)) may vary over time for a single participant and have been collected repeatedly throughout follow-up.
3. The statement, "If this were a multi-site study or one where the same individual was assessed multiple times for an outcome [...] incorporating random effects or using a GEE model would be appropriate", appears to contradict the study design and reported results. According to the manuscript (line 104), participants were followed from 18 July 2018 to 31 December 2022. During this period, the same participants were followed over time, and several key characteristics (such as age, occupation, education level, marital status, residence, abnormal genital discharge in the past 3 months, and condom use) may have changed for a given participant over the four-year follow-up. This could be observed in the distribution of participant characteristics presented in Tables 1 and 2 (lines 266 and 289, respectively). Therefore, time-varying changes in these factors at the individual level could significantly influence HIV infection risk.

In light of the above, using a Poisson model with random effects or a marginal Poisson model using Generalized Estimating Equations (GEE) (with a random intercept and interaction terms with time for key variables) is both justified and methodologically appropriate for this study. Even if the results from this model was to remain similar to those currently presented, from a methodological standpoint, this approach better accounts for the longitudinal nature of the data and should be seriously considered by the authors.

**Lines 154 – 160:** Thank you to the authors for the clarifications provided in response to my previous Comment 7 regarding the sensitivity analysis in light of the high loss to follow-up rate (22%). For the sensitivity analyses, the authors may formulate assumptions (ideally informed by a review of the literature) and present the corresponding results. A plausible assumption, in the lack of evidence to suggest otherwise, would be that the HIV incidence among participants lost to follow-up is similar to that observed among those who completed follow-up.

**Lines 210 – 215:** Thank you for these clarifications following my previous Comment 8 and for the additional information provided in the footnote of Table 1. However, I am somewhat confused by the description: "§Other includes (multiple options allowed): Professional/technical worker (19%), sales/service worker (24%), subsistence agricultural worker (16%), craft and related trades worker (15%), house helper/labourer (16%), motorcyclist (8%), unemployed (7%), etc.; N/A, not applicable." The sum of these percentages is 105%, and this is without considering the "etc." category. Could there be an error in these calculations? If so, please correct accordingly.

Additionally, reporting the HIV incidence within each of these subgroups would be valuable for readers.

**Lines 161 – 162:** Thank you to the authors for including the HIV diagnostic algorithm (S1 Fig). However, after reviewing it, I found the flow diagram somewhat confusing, particularly regarding the classification of positive and inconclusive cases. According to the algorithm, a participant is declared HIV-positive after reactive results on both the Determine and Stat-Pak tests. However, there is an arrow that subsequently leads to a "reactive" outcome followed by an "inconclusive" classification. This appears to be a misalignment or error in the linkage of results and arrows within the figure. I kindly ask the authors to review and correct this accordingly.

In addition, I would like to raise the following questions for clarification:

- Is the tie-breaker test (SD Bioline) routinely supplied by the Ministry of Health and available in all health facilities to ensure consistent implementation of this algorithm?
- As part of quality assurance procedures, is a second laboratory technician required to independently repeat the algorithm for a participant before an HIV-positive result is confirmed?

It would be helpful if the authors could clarify and include these details in the manuscript.

**Lines 210-218:** Thank you to the authors for including Supporting Table 2 in response to my previous Comment 27. As previously noted in table 1 (Line 266), the total percentage exceeds 100% for the "Other" occupation category: "¶Other includes (multiple options allowed): Professional/technical worker (19%), sales/service worker (24%), subsistence agricultural worker (16%), craft and related trades worker (15%), house helper/labourer (16%), motorcyclist (8%), unemployed (7%), etc."

Please review and correct this accordingly.

**Line 244 Fig 1:** Thank you for this clarification regarding my previous Comment 29, which reads as follows:

*"We have provided a detail to distinguish between the cohort and the trial in the methods section as written below: The main aim of the cohort study was to identify HIV-negative adults at high risk of HIV infection and prepare them for participation in a phase IIb HIV prophylactic vaccine trial (NCT04066881) at sites in Mozambique, South Africa, Tanzania, and Uganda. The trial aimed to determine the efficacy of two HIV prophylactic vaccine regimens and to compare the effectiveness of Truvada and Descovy as PrEP (Joseph, Kaleebu et al. 2019)."*

Does this imply that the 30 HIV-negative participants who completed the cohort study but were not be enrolled in the PrEPVacc trial were no longer considered to be at high risk of HIV infection? If so, this seems somewhat surprising, given that being at high risk of HIV acquisition was an inclusion criterion for enrollment in the cohort study.

I recommend that the authors add a footnote to Figure 1 to clarify this subgroup (N = 30) and explain the reason for their non-enrollment in the PrEPVacc trial.
