# Supplementary material for: Estimating HIV incidence and assessing associated risk factors among adults: Evidence from the 2018–2022 HIV vaccine preparedness cohort in Masaka, Uganda
Source: PLoS One. 2026 May 8;21(5):e0348769. doi: 10.1371/journal.pone.0348769 (PMC13155609; doi:10.1371/journal.pone.0348769)
Supplement: Attachment — Submitted filename: My Comments to Authors 06 Dec 2024.docx [file pone.0348769.s005.docx]

**Reviewing of the paper titled**

**HIV incidence and associated risk factors among adults enrolled in an HIV vaccine preparedness cohort in Masaka, Uganda**

Thank you for the opportunity to review this article, which has the potential to make a significant contribution to HIV research, particularly in the context of the "First 95" initiative. The article presents a study conducted as part of the PrEPVacc registration cohort study, aimed at estimating HIV incidence and identifying associated risk factors within a high-risk population in preparation for the PrEPVacc HIV vaccine trial. Conducted between 2018 and 2022 across multiple clinical research sites in East Africa, the study focuses particularly on Masaka, Uganda.

While the study offers valuable insights into HIV incidence and risk factors, there are several major flows that need to be addressed. These concerns primarily relate to the identification of recent HIV infections, study methodology, and the interpretation of certain results.

I set out my concerns in greater detail below.

**Major comments**

**Introduction**

The introduction begins with a compelling statistic, underlining the urgency of HIV prevention efforts, particularly in regions like Eastern and Southern Africa, which account for a significant proportion of new infections. The discussion of key populations and their disproportionate burden of HIV provides a well-justified rationale for targeting high-risk groups in research. However, there are areas where the introduction could be further refined to strengthen its impact and clarity within an epidemiological context.

Lines 67 – 71: Although the authors mention that current HIV incidence data are limited, they do not specify the scope or nature of these gaps. For instance, it would strengthen the introduction if the authors detailed regional disparities or methodological challenges in existing incidence data, particularly in populations similar to those studied.

Lines 67 – 79: In the introduction of this manuscript, the extensive discussion on the challenges associated with implementing clinical trials for prevention interventions, such as PrEP or HIV vaccines, appears unnecessary. It would be more appropriate for the authors to condense this section to maintain focus and conciseness.

Moreover, authors should briefly outline methodological innovations that address these challenges. This would provide a forward-looking perspective and demonstrate the study’s contribution to advancing HIV prevention research.

Lines 80 – 85 : Authors should :

- Clearly outline what sets this study apart from previous research.
- Justify the study's geographic focus with specific local epidemiological data.
- Include a concise literature review on HIV incidence and risk factors in Masaka to underscore the study's relevance and originality.

**Methods and Material**

Line 141 – 143 : How were participants in seroconversion identified? The use of the national screening algorithm based on rapid tests may not be sufficient, as it could yield false negatives. Therefore, it is necessary to conduct more advanced laboratory testing, such as recent infection tests (within a specific timeframe, e.g., 12 months), to confirm that the cases reported during follow-up are indeed new infections and not pre-existing ones.

Lines 145 – 147: This approach of analysis does not seem ideal given the study's cohort design. The major limitation of this approach is that it considers only inter-individual variability while completely ignoring intra-individual variability across all factors influencing the occurrence of new HIV infections. This effectively reduces the cohort study design to two cross-sectional studies, which is not appropriate.

We therefore recommend that the authors, instead of using two univariable and multivariable Poisson regression models, consider using a Poisson model with random effects or a marginal Poisson model with a Generalized Estimating Equations (GEE) approach. The working correlation matrix should be exchangeable, determined by the information criterion (QICu). Since the authors use STATA 18.0, we recommend that they review the documentation and utilize the **xtpoisson** command, which supports the implementation of these models.

Some critical subsections appear to be missing in the methodology:

- A detailed description of the study site.
- The sampling method employed within the community.
- The calculation of the sample size required to measure incidence in this population.
- A more precise description of the PrePVacc trial, including the primary and secondary endpoints.

**Results**

Lines 173 – 178: A substantial proportion of participants (22%) did not attend any follow-up visits, and only 38% completed the study. These figures raise concerns about potential attrition bias. The reasons for dropout are mentioned but not explored in depth. Authors should Perform a sensitivity analysis to assess the impact of attrition on the results and provide a detailed discussion of measures taken to minimize dropout.

Lines 224 : Table 1: Concerns Regarding the Categorization of the Variable "Occupation".

I have significant concerns about the categorization of the variable Occupation. The "Other" category encompasses 30% of the enrolled population (330 participants), which is an unusually high proportion. Additionally, this category includes diverse groups such as professional/technical workers, sales/service workers, office clerks, and students, which introduces considerable heterogeneity. The behaviors and HIV infection risks of students, for instance, are likely to differ significantly from those of professional or technical workers. Aggregating these distinct subgroups into a single category is methodologically inappropriate.

The use of this aggregated category in the analysis—and particularly in the interpretation of other groups, as observed on line 211—raises concerns about the validity and specificity of the findings.

We strongly recommend that the authors disaggregate this variable into more meaningful and homogeneous subcategories and reconsider its inclusion in the analyses to ensure more accurate and interpretable results. Using this aggregated category in the analysis, and particularly in the interpretation of other groups (as done on line 211), is therefore problematic.

We strongly recommend that the authors refine this variable by disaggregating its components and reconsider its inclusion in the analyses.

Line 246 : Table 2: The authors have not considered certain variables from the analysis in Table 1, such as marital status or occupation, which could have changed over the 4-year follow-up period and, consequently, influenced the risk of HIV infection. If the authors assessed these factors at each visit, we recommend treating these variables as time-dependent in the random effects Poisson model or the marginal Poisson model, as we suggested earlier.

**Discussion**

Line 253: The authors mention low PrEP uptake in the study population as a potential reason for persistent HIV transmission. While this is an important observation, it would be valuable to delve deeper into why PrEP uptake is low. Are there barriers to access (e.g., cost, stigma, lack of awareness), or are there other socio-behavioral factors? This could inform targeted interventions to improve PrEP coverage and adherence.

Line 260 – 265: The discussion largely focuses on descriptive findings but could benefit from more in-depth exploration of the theoretical mechanisms underlying the observed associations. For example, the authors could expand on how specific socio-economic and cultural factors (e.g., gender inequality, lack of access to healthcare) might exacerbate HIV transmission, particularly in subpopulations like women and residents of fishing communities.

Lines 292 – 294 : The authors briefly mention the limitations of the data, such as selection bias due to participant dropout and the potential for underreporting of HIV risk factors. However, the statistical methods used to handle these issues are not discussed in detail. It would be beneficial for the authors to address whether any sensitivity analyses were performed to account for potential biases or missing data, or if any imputation techniques were used.

**Conclusion**

Line 301 – 303 : The discussion on prevention strategies is relevant, but it could be enhanced by offering more concrete recommendations for public health interventions. For example, given the identified risk factors (e.g., low PrEP uptake, alcohol and drug use), the authors could discuss specific interventions that could address these issues within the study population or similar high-risk groups.

Line 306: While the authors suggest exploring innovative methodologies for future trials, they could clarify what types of methodologies they consider to be the most promising. For example, what new approaches could be explored to assess the efficacy of interventions, especially in populations that have traditionally been harder to reach? Specific examples of innovative methodologies (e.g., digital health tools, community-based interventions) would make this recommendation more actionable.

**Minor comments**

**Title:**

I suggest the title: “*Estimating HIV incidence and assessing associated risk factors among adults: Evidence from the 2018 HIV vaccine preparedness cohort in Masaka, Uganda*”

**Introduction**

Lines 58-59 : Integrate updated HIV epidemiological statistics to reflect the most recent trends (2023) and provide an overview of the evolution over the past three decades. The authors are encouraged to reference **THE 2024 GLOBAL AIDS UPDATE** report.

Lines 67 – 69 : Authors should support this statement with evidence (figures).

**Methods and Material**

Lines 103 – 106 : The reliance on HIV counseling and testing (HCT) outreach programs for recruitment may introduce selection bias by disproportionately including individuals already engaged with healthcare services. This could limit the generalizability of the findings to the broader at-risk population. Authors should include a discussion of the potential biases introduced by the recruitment strategy and consider alternative methods for reaching less-engaged populations.

Lines 107-108 : The description of screening and enrollment procedures is concise but lacks detail on how potential participants were approached and convinced to join the study.

Lines 121 – 126: The sentence is too long. It would be preferable for the authors to present the complete participation criteria of the study using bullet points.

Line 129: The authors should also provide evidence or studies on the intrinsic performance metrics of the screening algorithm in their country, including sensitivity, specificity, and, most importantly, the positive and negative predictive values, to reassure readers about the reliability of the results.

Lines 129 – 134: The description of laboratory procedures, while adequate, could benefit from more detail on quality control measures for HIV testing and pregnancy screening to ensure reproducibility. Authors should Provide detailed information on quality assurance practices, including, where applicable, external quality control and validation or inter-laboratory comparisons.

Line 134: For enhanced clarity, the authors should present the HIV screening and testing algorithm using a diagram.

Lines 135 – 136: What is the outcome for pregnant women in the study? The authors should clarify the follow-up procedures specifically for pregnant women!

Lines 150 – 154 : The methods for collecting data on HIV risk behaviors rely on self-reports, which are susceptible to memory bias and social desirability bias. Discuss these limitations explicitly and, if available, include validation measures (e.g., biomarkers or triangulation) to corroborate self-reported data.

Line 159 – 160 : The authors should justify why they decided to include age and gender a priori in the multivariable model.

**Results**

Lines 178 -181 : The authors should provide a table or figure to effectively support these results.

Line 202 : Fig 1 : The authors should provide a more explicit explanation for the exclusion reason labeled as "investigator discretion" and include it under the "Other" category.

Line 199 Fig 1 : Here, the authors present 370 participants who completed the cohort study and will be enrolled in the PrePVacc trial, along with 30 participants who completed the study but will not be enrolled. Could the authors clarify the distinction between this cohort study and the PrePVacc trial? If the two studies are independent, why is the PrePVacc trial mentioned in this context?

Line 208 : If the 24 new infections were identified using rapid HIV diagnostic tests, this is insufficient to draw definitive conclusions, as these tests do not confirm whether the infections are indeed new. Additional recent HIV infection assays should be conducted. If the authors are unable to perform such tests, they must acknowledge this limitation in the discussion.

Line 209 – 221 : Here, the authors interpret the results of the model based on baseline factors. However, the interpretation includes both univariable and multivariable analyses, leading to redundancy (e.g., for the variable "Sex"). Generally interpretation focuses on the results of the multivariable analysis, as it accounts for adjustments across other factors, thereby providing a more accurate representation of the phenomenon in the study population, where these factors operate simultaneously.

Lines 224 : Table 1: Could the authors explain the "N/A" for the variable "Circumcised" in both the univariable and multivariable analyses?

**Discussion**

Line 256 : While the authors present the high incidence of HIV and its association with certain risk factors, the discussion could benefit from a more nuanced exploration of why the incidence rate was "lower than expected" (line 256). Moreover, the authors should specified the expected incidence rate.

Lines 282 – 291: The authors note that abnormal genital discharge was unexpectedly associated with a reduced risk of HIV acquisition. While they mention potential misclassification or confounding factors, it would be helpful to further elaborate on how other unmeasured variables (e.g., hormonal fluctuations, non-STI-related vaginal infections) might explain this result. A more detailed exploration of potential confounders would enhance the robustness of the interpretation.

Lines 295 – 298: The authors mention that HIV risk factors were self-reported, which is a common limitation in cohort studies. While this is acknowledged, the authors could strengthen the discussion by elaborating on how these biases (e.g., recall bias, social desirability bias) could specifically affect their findings. Furthermore, it would be useful to consider how the inclusion of biomarkers or objective measures of risk (e.g., STI testing) could have enhanced the reliability of their data.

**Conclusion**

Lines 303 – 305: The authors could further explore how the findings of this study could influence public health policies and programs. For example, how might public health systems prioritize HIV prevention in high-risk populations? Expanding on this point would provide a clearer path from research findings to practical, policy-oriented action.
