# Supplementary material for: Estimating HIV incidence and assessing associated risk factors among adults: Evidence from the 2018–2022 HIV vaccine preparedness cohort in Masaka, Uganda
Source: PLoS One. 2026 May 8;21(5):e0348769. doi: 10.1371/journal.pone.0348769 (PMC13155609; doi:10.1371/journal.pone.0348769)
Supplement: Attachment — Submitted filename: Response to Reviewers_Final8March2025.docx [file pone.0348769.s006.docx]

**Response sheet for submission ID PONE-D-24-44704 R1: HIV incidence and associated risk factors among adults enrolled in an HIV vaccine preparedness cohort in Masaka, Uganda**

**Comments and responses**

We wish to thank the reviewers for their valuable comments. Please find below a point-by-point response to comments raised.

**Reviewer #1**

**Comment 1**

Thank you for the opportunity to review this article, which has the potential to make a significant contribution to HIV research, particularly in the context of the "First 95" initiative. The article presents a study conducted as part of the PrEPVacc registration cohort study, aimed at estimating HIV incidence and identifying associated risk factors within a high-risk population in preparation for the PrEPVacc HIV vaccine trial. Conducted between 2018 and 2022 across multiple clinical research sites in East Africa, the study focuses particularly on Masaka, Uganda.

While the study offers valuable insights into HIV incidence and risk factors, there are several major flows that need to be addressed. These concerns primarily relate to the identification of recent HIV infections, study methodology, and the interpretation of certain results. I set out my concerns in greater detail below.

**Major comments**

**Comment 2:** The introduction begins with a compelling statistic, underlining the urgency of HIV prevention efforts, particularly in regions like Eastern and Southern Africa, which account for a significant proportion of new infections. The discussion of key populations and their disproportionate burden of HIV provides a well-justified rationale for targeting high-risk groups in research. However, there are areas where the introduction could be further refined to strengthen its impact and clarity within an epidemiological context.

Lines 67 – 71: Although the authors mention that current HIV incidence data are limited, they do not specify the scope or nature of these gaps. For instance, it would strengthen the introduction if the authors detailed regional disparities or methodological challenges in existing incidence data, particularly in populations similar to those studied.

**Response:** We appreciate the reviewer for their suggestion. We have revised the introduction to highlight the disparities in HIV incidence within and between different key populations in sub-Saharan Africa.

**Comment 3:** Lines 67 – 79: In the introduction of this manuscript, the extensive discussion on the challenges associated with implementing clinical trials for prevention interventions, such as PrEP or HIV vaccines, appears unnecessary. It would be more appropriate for the authors to condense this section to maintain focus and conciseness.

Moreover, authors should briefly outline methodological innovations that address these challenges. This would provide a forward-looking perspective and demonstrate the study’s contribution to advancing HIV prevention research.

**Response**: We thank the reviewer for this insight. We have included details on methodological innovations and recommendations that address challenges around estimating HIV incidence in HIV prevention trials, namely: adjusting for pre-existing infections, product adherence, and discontinuation in sample size calculations, while also developing biomarkers and alternative trial designs.

**Comment 4:** Lines 80 – 85: Authors should:

- Clearly outline what sets this study apart from previous research.
- Justify the study's geographic focus with specific local epidemiological data.
- Include a concise literature review on HIV incidence and risk factors in Masaka to underscore the study's relevance and originality.

**Response:** We thank the reviewer for their suggestions. The introduction has been revised accordingly.

**Methods and Material**

**Comment 5:** Line 141 – 143: How were participants in seroconversion identified? The use of the national screening algorithm based on rapid tests may not be sufficient, as it could yield false negatives. Therefore, it is necessary to conduct more advanced laboratory testing, such as recent infection tests (within a specific timeframe, e.g., 12 months), to confirm that the cases reported during follow-up are indeed new infections and not pre-existing ones.

**Response:** As outlined in the manuscript's introduction, the primary aim of the PrEPVacc registration cohort was to estimate HIV incidence in populations targeted for the PrEPVacc trial. Only individuals who tested HIV-negative at baseline and met the eligibility criteria were enrolled. Those who tested HIV-positive were ineligible and were referred for HIV care. Recency testing was not performed, as the study did not aim to distinguish newly acquired HIV infections from prevalent cases.

HIV testing was at screening, enrollment, and at quarterly follow-up visits was performed in accordance with Uganda’s national guidelines, using the Serial HIV Testing Algorithm for individuals above 18 months of age. This algorithm has a sensitivity of 99.2%, specificity of 99.1%, positive predictive value of 99.0% and negative predictive value of 99.2% (Kaleebu, Kitandwe et al. 2018).

To maintain quality control, study staff underwent training and certification by a national provider to conduct HIV testing services. Internal quality control was performed before testing participant samples, and the study site participated in Uganda’s national external quality assurance (EQA) program, with EQA conducted quarterly. A section detailing these quality control procedures has been added to the revised manuscript under laboratory procedures.

**Comment 6:** Lines 145 – 147: This approach of analysis does not seem ideal given the study's cohort design. The major limitation of this approach is that it considers only inter-individual variability while completely ignoring intra-individual variability across all factors influencing the occurrence of new HIV infections. This effectively reduces the cohort study design to two cross-sectional studies, which is not appropriate.

We therefore recommend that the authors, instead of using two univariable and multivariable Poisson regression models, consider using a Poisson model with random effects or a marginal Poisson model with a Generalized Estimating Equations (GEE) approach. The working correlation matrix should be exchangeable, determined by the information criterion (QICu). Since the authors use STATA 18.0, we recommend that they review the documentation and utilize the **xtpoisson** command, which supports the implementation of these models.

Some critical subsections appear to be missing in the methodology:

- A detailed description of the study site.
- The sampling method employed within the community.
- The calculation of the sample size required to measure incidence in this population.
- A more precise description of the PrePVacc trial, including the primary and secondary endpoints.

**Response:** We appreciate the reviewer’s detailed comment. The analytical approach used in this paper is a standard method for analyzing incidence data from a single study site. Similar approaches have been applied in other studies (Ahmed, Lutalo et al. 2001, De Schacht, Mabunda et al. 2014, Westreich, Jamal et al. 2014, Gómez-Olivé, Houle et al. 2020).

If this were a multi-site study or one where the same individual was assessed multiple times for an outcome (e.g., repeated blood pressure measurements), incorporating random effects or using a GEE model would be appropriate. However, in our case, each participant had a single outcome (HIV status at the end of follow-up), and the study was conducted at a single site (Masaka). Therefore, there was no intra-individual variability or clear random effect to account for.

We have also added separate subsections in the methodology section for study design, study site, and population, outlining the sample selection process. Additionally, we have provided a more detailed description of the PrEPVacc trial in the revised manuscript.

For the sample size, a post hoc sample size calculation showed ≥900 person-years of follow-up (PYO), and an incidence rate of ~2.7/100PYO provided a confidence interval with a precision ≤3/ 100PYO. Additionally, with 900 person years of follow up, we had ≥80% power to identify factors with an incident rate ratio ≥2.5.

**Results**

**Comment 7:** Lines 173 – 178: A substantial proportion of participants (22%) did not attend any follow-up visits, and only 38% completed the study. These figures raise concerns about potential attrition bias. The reasons for dropout are mentioned but not explored in depth. Authors should perform a sensitivity analysis to assess the impact of attrition on the results and provide a detailed discussion of measures taken to minimize dropout.

**Response**: Thank you for this comment. We acknowledge that the considerable attrition in this study may have introduced bias in the observed incidence rate, and we have explicitly addressed this limitation in the manuscript. A separate analysis on LTFU in this cohort (Kabarambi, Kansiime et al. 2022) found that individuals at potentially higher risk of HIV—including young people (<25 years), females, recreational drug users, and those engaged in transactional sex—were more likely to be lost to follow-up. This suggests that our HIV incidence estimate may be an underestimate.

Additionally, participant follow-up was significantly impacted by disruptions caused by COVID-19. However, we do not consider a sensitivity analysis necessary, as it would require hypothetical assumptions about incidence among those lost to follow-up. Instead, we have clearly acknowledged this limitation and detailed the measures taken in the study to minimize dropout.

**Comment 8**: Lines 224: Table 1: Concerns Regarding the Categorization of the Variable "Occupation".

I have significant concerns about the categorization of the variable Occupation. The "Other" category encompasses 30% of the enrolled population (330 participants), which is an unusually high proportion. Additionally, this category includes diverse groups such as professional/technical workers, sales/service workers, office clerks, and students, which introduces considerable heterogeneity. The behaviors and HIV infection risks of students, for instance, are likely to differ significantly from those of professional or technical workers. Aggregating these distinct subgroups into a single category is methodologically inappropriate.

The use of this aggregated category in the analysis—and particularly in the interpretation of other groups, as observed on line 211—raises concerns about the validity and specificity of the findings.

We strongly recommend that the authors disaggregate this variable into more meaningful and homogeneous subcategories and reconsider its inclusion in the analyses to ensure more accurate and interpretable results. Using this aggregated category in the analysis, and particularly in the interpretation of other groups (as done on line 211), is therefore problematic.

We strongly recommend that the authors refine this variable by disaggregating its components and reconsider its inclusion in the analyses.

**Response**: Thank you for this comment. We appreciate the rationale behind your suggestion. However, the categorization of the occupation variable was based on the known HIV epidemiology in our study setting. Sex workers, salon/bar/lodge workers, and fisher-folk have consistently been identified as populations at exceptionally high risk of HIV acquisition. In contrast, while other occupations, encompass diverse groups (e.g., house helps vs. professional/technical workers); they have not been specifically highlighted as high-risk groups in the same way. Indeed, in our analysis, this group has the lowest HIV incidence. In the revised manuscript, we provide a breakdown of the occupations in this group in a footer under Table 1.

**Comment 9:** Line 246: Table 2: The authors have not considered certain variables from the analysis in Table 1, such as marital status or occupation, which could have changed over the 4-year follow-up period and, consequently, influenced the risk of HIV infection. If the authors assessed these factors at each visit, we recommend treating these variables as time-dependent in the random effects Poisson model or the marginal Poisson model, as we suggested earlier.

**Response**: We agree with the reviewer on this comment; however, these data were only collected at baseline. As such, we are unable to account for changes during follow-up

**Discussion**

**Comment 10:** Line 253: The authors mention low PrEP uptake in the study population as a potential reason for persistent HIV transmission. While this is an important observation, it would be valuable to delve deeper into why PrEP uptake is low. Are there barriers to access (e.g., cost, stigma, lack of awareness), or are there other socio-behavioral factors? This could inform targeted interventions to improve PrEP coverage and adherence.

**Response**: We thank the reviewer for their observation and concur with their view. The reasons for low uptake have been studied and published using data from the same cohort (Kusemererwa, Kansiime et al. 2021). Briefly, uptake was low in this population mainly because of fear of pill burden, stigma, and participants needing more time to decide to start oral PrEP. We have included a sentence in the revised manuscript summarizing the key reasons for non-uptake.

**Comment 11:** Line 260 – 265: The discussion largely focuses on descriptive findings but could benefit from more in-depth exploration of the theoretical mechanisms underlying the observed associations. For example, the authors could expand on how specific socio-economic and cultural factors (e.g., gender inequality, lack of access to healthcare) might exacerbate HIV transmission, particularly in subpopulations like women and residents of fishing communities.

**Response**: We appreciate the reviewer for their suggestion. In the revised manuscript, we have expanded our discussion on the of gender dynamics and access to healthcare in shaping HIV risk. Additionally, we had already provided some detail on the factors influencing HIV risk among residents of fishing communities in paragraph 3.

**Comment 12**: Lines 292 – 294: The authors briefly mention the limitations of the data, such as selection bias due to participant dropout and the potential for underreporting of HIV risk factors. However, the statistical methods used to handle these issues are not discussed in detail. It would be beneficial for the authors to address whether any sensitivity analyses were performed to account for potential biases or missing data, or if any imputation techniques were used.

**Response**: We thank the reviewer for this comment. As noted in our response to comment 6, we have acknowledged the limitations associated with high attrition and have referenced a published paper on loss to follow up in our cohort (Kabarambi, Kansiime et al. 2022). We have also explained why we did not perform sensitivity analyses.

**Conclusion**

**Comment 13:** Line 301 – 303: The discussion on prevention strategies is relevant, but it could be enhanced by offering more concrete recommendations for public health interventions. For example, given the identified risk factors (e.g., low PrEP uptake, alcohol and drug use), the authors could discuss specific interventions that could address these issues within the study population or similar high-risk groups.

**Response**: We thank the reviewer for their suggestion. In the revised manuscript, we recommend targeted HIV education and outreach programs as way of enhancing access to oral PrEP in fishing communities through addressing stigma and logistical barriers. Additionally, counselling and support groups can play a crucial role in mitigating substance use-related risks and encouraging PrEP uptake.

**Comment 14**: Line 306: While the authors suggest exploring innovative methodologies for future trials, they could clarify what types of methodologies they consider to be the most promising. For example, what new approaches could be explored to assess the efficacy of interventions, especially in populations that have traditionally been harder to reach? Specific examples of innovative methodologies (e.g., digital health tools, community-based interventions) would make this recommendation more actionable.

**Response**: We thank the reviewer for this valuable suggestion. The reason we suggest exploration of innovative methodologies for future HIV prevention trials is because the use of placebo-controlled trials is becoming less feasible due to the increasing availability of highly effective HIV prevention tools. In the revised manuscript, we have provided some of the proposed approaches to address this problem. These include: r**egistration cohorts**, which estimate HIV incidence in real-world settings without the experimental intervention; **recency assays**, which estimate recent HIV infection rates at the time of trial screening; **external trial placebo arms**, where data from previous placebo-controlled HIV prevention trials serve as a reference for HIV incidence; **biological markers of HIV incidence**, used to gauge an individual’s HIV exposure and infer intervention efficacy; **drug concentration measurements**, which assess how effectively a treatment prevents HIV based on pharmacokinetics; and **immune biomarkers**, such as antibodies, to predict the protective effectiveness of new prevention strategies.(Donnell, Kansiime et al. 2023)

**Minor comments**

**Comment 15: Title:** I suggest the title: “*Estimating HIV incidence and assessing associated risk factors among adults: Evidence from the 2018-2022 HIV vaccine preparedness cohort in Masaka, Uganda*”

**Response**: We thank the reviewer for their suggestion. The title has been revised accordingly.

**Introduction**

**Comment 16:** Lines 58-59: Integrate updated HIV epidemiological statistics to reflect the most recent trends (2023) and provide an overview of the evolution over the past three decades. The authors are encouraged to reference **THE 2024 GLOBAL AIDS UPDATE** report.

**Response:** We thank the reviewer for this suggestion. The introduction has been updated to reflect the most recent trends, as referenced.

**Comment 17**: Lines 67 – 69: Authors should support this statement with evidence (figures).

**Response:** We have revised the manuscript to include evidence.

The line now reads: *…Global HIV incidence is declining, with fewer new infections reported in 2023 than at any point since the mid-1990s (UNAIDS 2024). The most significant reductions have occurred in sub-Saharan Africa, where new infections among adults aged 15–49 dropped by 54%, from 1.1 million in 2010 to 510,000 in 2022…..*

**Methods and Material**

**Comment 18:** Lines 103 – 106: The reliance on HIV counseling and testing (HCT) outreach programs for recruitment may introduce selection bias by disproportionately including individuals already engaged with healthcare services. This could limit the generalizability of the findings to the broader at-risk population. Authors should include a discussion of the potential biases introduced by the recruitment strategy and consider alternative methods for reaching less-engaged populations.

**Response:** We would like to clarify that our recruitment strategy was not limited to outreach programs. We also employed a door-to-door approach to reach individuals who may not have engaged with health facilities, including testing points in areas where transactional sex occurred. Additional details have been included in the Methods section under "Study population and recruitment"

**Comment 19**: Lines 107-108: The description of screening and enrollment procedures is concise but lacks detail on how potential participants were approached and convinced to join the study.

**Response:** We have added a separate section under “Study population and recruitment” to describe how potential participants were recruited from the community and referred to the research site for screening.

**Comment 20:** Lines 121 – 126: The sentence is too long. It would be preferable for the authors to present the complete participation criteria of the study using bullet points.

**Response:** We appreciate the reviewer for this comment. The sentence has been revised with the criteria broken down using commas and Roman numerals and now appears as:

*A participant completed study follow-up when: i) they enrolled in the PrEPVacc trial, ii) withdrew consent, iii) tested HIV-positive, iv) were determined to be ineligible for continued follow-up based on a re-assessment of his/her risk and study eligibility or v) were non-compliant to the study procedures or when the PrEPVacc trial was fully enrolled.*

**Comment 21:** Line 129: The authors should also provide evidence or studies on the intrinsic performance metrics of the screening algorithm in their country, including sensitivity, specificity, and, most importantly, the positive and negative predictive values, to reassure readers about the reliability of the results.

**Response:** We appreciate the reviewer’s suggestion. The screening algorithm in the country has a sensitivity of 99.2%, specificity of 99.1%, and positive predictive value of 99.0% and a negative predictive value of 99.2% (Kaleebu, Kitandwe et al. 2018)(Kaleebu et al, 2018). These values have been included in the revised manuscript.

**Comment 22:** Lines 129 – 134: The description of laboratory procedures, while adequate, could benefit from more detail on quality control measures for HIV testing and pregnancy screening to ensure reproducibility. Authors should Provide detailed information on quality assurance practices, including, where applicable, external quality control and validation or inter-laboratory comparisons.

**Response:** We appreciate the reviewer for their suggestion. A section on quality control measures has been included under the laboratory procedures section in the revised manuscript.

**Comment 23:** Line 134: For enhanced clarity, the authors should present the HIV screening and testing algorithm using a diagram.

**Response**: We thank the reviewer for this suggestion. We reference the national guidelines that include a diagram for the testing algorithm. The national algorithm diagram has been included as a supporting document (S1_Figure).

*Supporting Figure 1: Serial HIV testing algorithm for persons above 18 months of age*

**Comment 24:** Lines 135 – 136: What is the outcome for pregnant women in the study? The authors should clarify the follow-up procedures specifically for pregnant women!

**Response:** All those who tested positive for pregnancy were referred for antenatal care. Those who tested positive for pregnancy and HIV positive were also referred for prevention of mother-to-child transmission services. Details have been included in the manuscript under study procedures.

Information on pregnancy outcomes are also provided in another manuscript (Kusemererwa, Kansiime et al. 2024).

**Comment 25:** Lines 150 – 154: The methods for collecting data on HIV risk behaviors rely on self-reports, which are susceptible to memory bias and social desirability bias. Discuss these limitations explicitly and, if available, include validation measures (e.g., biomarkers or triangulation) to corroborate self-reported data.

**Response:** We thank the reviewer for this suggestion. We discuss this limitation and have also included an explanation on how the biases were addressed. We have also acknowledged that objective measures, such as STI testing, would have provided valuable biological confirmation of HIV risk.

*To mitigate these biases, multiple questions were used to verify and crosscheck responses. Use of objective measures, such as STI testing, would have provided valuable biologically confirmation of HIV risk, though this was not feasible in the current study.*

**Comment 26:** Line 159 – 160: The authors should justify why they decided to include age and gender a priori in the multivariable model.

**Response:** We wish to clarify that age and gender were included a priori based on established evidence from previous research, which consistently identifies them as major key risk factors for incident HIV(Dellar, Dlamini et al. 2015, Kharsany, Cawood et al. 2019, Stannah, Soni et al. 2023). Their inclusion ensures that the analysis accounts for known demographic influences on HIV risk, enhancing the model’s robustness and comparability with existing studies.

**Results**

**Comment 27:** Lines 178 -181: The authors should provide a table or figure to effectively support these results.

**Response:** A table has been provided supporting document 2_Table as shown below:

**Supporting Table 1:** **Comparison of baseline characteristics between participants who did not attend any follow-up visit and those who did in a HIV vaccine preparedness study at Masaka, Uganda.**

| **Characteristics** | **All enrolled** | **≥1 follow-up visit** | **No follow-up visits** | **P-value** |
| --- | --- | --- | --- | --- |
|  | **N (%)^¥^** | **n (%)^¥^** | **n (%)^¥^** |  |
| **Total** | **1422 (100)** | **1115 (78)** | **307 (22)** |  |
| **Gender** |  |  |  | P<0.001 |
| Male | 368 (26) | 346 (31) | 22 (7) |  |
| Female | 1054 (74) | 769 (69) | 285 (93) |  |
| **Age (years)** |  |  |  | 0.251 |
| ≤24 | 791 (56) | 612 (55) | 179 (58) |  |
| >24 | 631 (44) | 503 (45) | 128 (42) |  |
| **Education** |  |  |  | 0.951 |
| ≤Primary | 862 (61) | 677 (61) | 185 (60) |  |
| ≥Secondary | 560 (39) | 438 (39) | 122 (40) |  |
| **Marital Status** |  |  |  | 0.375 |
| Single | 763 (54) | 595 (53) | 168 (55) |  |
| Married/cohabiting/relationship | 385 (27) | 310 (28) | 75 (24) |  |
| Divorced/ separated/ widowed | 274 (19) | 210 (19) | 64 (21) |  |
| **Religion** |  |  |  | 0.089 |
| Christian | 1068 (75) | 827 (74) | 241 (79) |  |
| Muslim/ other | 354 (25) | 288 (26) | 66 (21) |  |
| **Occupation** |  |  |  | P<0.001 |
| Other^¶^ | 375 (26) | 337 (30) | 38 (12) |  |
| Sex worker | 722 (51) | 509 (46) | 213 (69) |  |
| Salon/Lodge/Bar worker | 230 (16) | 181 (16) | 49 (16) |  |
| Subsistence fisheries worker | 95 (7) | 88 (8) | 7 (2) |  |
| **Residence** |  |  |  | P<0.001 |
| Non-fishing village | 1216 (86) | 928 (83) | 288 (94) |  |
| Fishing village | 206 (14) | 187 (17) | 19 (6) |  |
| **Had transactional sex in the last 3 months** |  |  |  | P<0.001 |
| No | 137 (10) | 129 (12) | 8 (3) |  |
| Yes | 1285 (90) | 986 (88) | 299 (97) |  |
| **Number of partners last 3 months** |  |  |  | 0.017 |
| ≤5 | 523 (37) | 428 (38) | 95 (31) |  |
| ≥6 | 899 (63) | 687 (62) | 212 (69) |  |

**^¥^**^Column percentages; ¶Other includes (multiple options allowed): Professional/technical worker (19%), sales/service worker (24%), subsistence agricultural worker (16%), craft and related trades worker (15%), house helper/labourer (16%), motorcyclist (8%), unemployed (7%), etc; §Formal/informal employment, family, spouse etc.^

**Comment 28:** Line 202: Fig 1: The authors should provide a more explicit explanation for the exclusion reason labeled as "investigator discretion" and include it under the "Other" category.

**Response:** We have clarified in the Figure 1 *that “Investigator discretion includes any condition(s) such as inability to provide informed consent, alcohol dependence, or cognitive impairment that, in the opinion of the investigator, might make it difficult for an individual to participate in the study or for the study to achieve its objectives.”*

**Comment 29:** Line 199 Fig 1: Here, the authors present 370 participants who completed the cohort study and will be enrolled in the PrEPVacc trial, along with 30 participants who completed the study but will not be enrolled. Could the authors clarify the distinction between this cohort study and the PrePVacc trial? If the two studies are independent, why is the PrePVacc trial mentioned in this context?

**Response:** We have provided a detail to distinguish between the cohort and the trial in the methods section as written below:

*The main aim of the cohort study was to identify HIV-negative adults at high risk of HIV infection and prepare them for participation in a phase IIb HIV prophylactic vaccine trial (NCT04066881) at sites in Mozambique, South Africa, Tanzania, and Uganda The trial aimed to determine the efficacy of two HIV prophylactic vaccine regimens and to compare the effectiveness of Truvada and Descovy as PrEP(Joseph, Kaleebu et al. 2019).*

**Comment 30:** Line 208: If the 24 new infections were identified using rapid HIV diagnostic tests, this is insufficient to draw definitive conclusions, as these tests do not confirm whether the infections are indeed new. Additional recent HIV infection assays should be conducted. If the authors are unable to perform such tests, they must acknowledge this limitation in the discussion.

**Response:** We would like to clarify that the primary aim of the registration cohort was to identify individuals testing positive for HIV to estimate incidence rates, which informed the sample size calculation for the PrEPVacc trial, as detailed in the manuscript's introduction and methods sections.

Participants were required to test HIV-negative for enrollment in the cohort study and underwent testing every three months. Those who later tested HIV-positive were classified as newly infected, as they had tested negative at the study's onset.

According to the national guidelines (Uganda 2022), all individuals aged 15 years or older who are newly diagnosed with HIV through the national HIV testing algorithm should undergo recent infection testing.

Recency testing was not performed, as the study did not aim to distinguish newly acquired HIV infections from prevalent cases.

**Comment 31:** Line 209 – 221: Here, the authors interpret the results of the model based on baseline factors. However, the interpretation includes both univariable and multivariable analyses, leading to redundancy (e.g., for the variable "Sex"). Generally, interpretation focuses on the results of the multivariable analysis, as it accounts for adjustments across other factors, thereby providing a more accurate representation of the phenomenon in the study population, where these factors operate simultaneously.

**Response:** We wish to clarify that we presented results for both univariable and multivariable analyses, focusing our discussion on factors from the multivariable analysis that remained significantly associated with incident HIV.

**Comment 32:** Lines 224: Table 1: Could the authors explain the "N/A" for the variable "Circumcised" in both the univariable and multivariable analyses?

**Response:** We thank the reviewer for this observation. N/A has been defined in the footer of the table in the revised manuscript.

*N/A stands for not applicable*

**Discussion**

**Comment 33:** Line 256: While the authors present the high incidence of HIV and its association with certain risk factors, the discussion could benefit from a more nuanced exploration of why the incidence rate was "lower than expected" (line 256). Moreover, the authors should specified the expected incidence rate.

**Response:** Previous reports in similar populations showed HIV incidence rates ranging from 3.1 to 9.3 per 100PYs. In the revised manuscript, we attribute the lower HIV incidence in our study to the increasing access to a combination of effective interventions, including HCT, condom use, voluntary medical [male circumcision](https://www.sciencedirect.com/topics/medicine-and-dentistry/circumcision), the use of antiretroviral drugs for the prevention of mother-to-child transmission, PrEP, and treatment as prevention (Kasamba, Mugisha et al. 2023).

**Comment 34:** Lines 282 – 291: The authors note that abnormal genital discharge was unexpectedly associated with a reduced risk of HIV acquisition. While they mention potential misclassification or confounding factors, it would be helpful to further elaborate on how other unmeasured variables (e.g., hormonal fluctuations, non-STI-related vaginal infections) might explain this result. A more detailed exploration of potential confounders would enhance the robustness of the interpretation.

**Response:** We thank the reviewer for their insight. However, we have included the following detail to the discussion about vaginal discharge and risk of HIV acquisition. **“**Alternatively, the abnormal discharge could have resulted from a disruption of the normal vaginal flora rather than an STI. While vaginal discharge itself does not directly prevent HIV, a healthy vaginal microbiome, dominated by Lactobacillus, plays a key role in reducing HIV susceptibility by maintaining an acidic pH, forming a mucosal barrier, and preventing inflammation (Armstrong and Kaul 2021). Nevertheless, other studies have also found no association between abnormal vaginal discharge and an increased risk of HIV acquisition (Mlisana, Naicker et al. 2012).”

**Comment 35:** Lines 295 – 298: The authors mention that HIV risk factors were self-reported, which is a common limitation in cohort studies. While this is acknowledged, the authors could strengthen the discussion by elaborating on how these biases (e.g., recall bias, social desirability bias) could specifically affect their findings. Furthermore, it would be useful to consider how the inclusion of biomarkers or objective measures of risk (e.g., STI testing) could have enhanced the reliability of their data.

**Response:** We appreciate the reviewer’s suggestion. We have incorporated a discussion on how we minimized these biases, including the recommendation to use objective measures such as STI testing.

**Conclusion**

**Comment 36:** Lines 303 – 305: The authors could further explore how the findings of this study could influence public health policies and programs. For example, how might public health systems prioritize HIV prevention in high-risk populations? Expanding on this point would provide a clearer path from research findings to practical, policy-oriented action.

**Response:** We thank the reviewer for their suggestion. The conclusion has been revised accordingly.

**Reviewer #2**

This is an important paper from a study to determine HIV incidence in the post-PrEP scale-up era. The study is quite useful for planning HIV prevention studies, including vaccines. However, the study design and results need some clarification.

**Response:** We thank the reviewer for their comment on the importance of our paper.

**Comment 1**. The authors state that “Additionally, time-related predictors, including duration of follow-up and the calendar period which person-years were accrued”. This statement is unclear as the variables that were time-updated are not mentioned. Specifically, since sex behaviours (and other behaviours) change during the study these variables (e.g. sex with multiple partners, alcohol, STIs etc) could have been modelled as time-updated variables.

**Response:** Thank you for this comment. We wish to clarify that the “time-related predictors” that we were referring to were “duration of follow-up and calendar period”. As you have advised in comment 4 below, we have edited to remove the wording “time-related predictors”. To incorporate the potential change in risk behaviours over time after the baseline assessments, we conducted an additional analysis presented in Table 2 where the risk behaviour data collected at the last assessment in the study (or prior to sero-conversion if applicable) was used.

**Comment 2.** Please clarify whether all seroconverted patients were linked to care and started on antiretroviral therapy.

**Response:** We wish to confirm that all patients who sero-converted were linked to care.

**Comment 3**. Was PrEP made available to all eligible participants. By the Ugandan guidelines, all the participants are eligible for PrEP. It is also not clear whether these patients received basic HIV prevention health education. These issues should be discussed in the ethics section

**Response:** We thank the reviewer for their suggestion. We have included text in the section for procedures that information and counselling on PrEP was provided and those willing to initiate PrEP, referral was made to a PrEP provider. We have also included the same in the ethics section of the revised manuscript.

**Comment 4.** The use of “time-related variables” is confusing as it will be confused with time-updated variables. Please use words such as cohort year or number of follow-up years.

**Response**: Thank you for this comment. We have edited to remove the wording “time-related variables”.

**Comment 5**. The number of incident cases was few and a brief table with their characteristics (Age, sex, and follow-up time) would be helpful, especially as a supplementary table.

**Response**: The number of incident cases by sub-group, are included in tables 1 & 2, in the first column in the HIV incidence section.

**Comment 6**. The other limitation is that there was high LTFU and the number of events is few. As can be seen, the Confidence intervals are quite wide.

**Response:** We appreciate the reviewer’s feedback. As also noted by the first reviewer, we have acknowledged the high LTFU as a limitation and addressed it in the revised manuscript. We recognize that the considerable attrition in this study may have introduced bias in the observed incidence rate. A separate paper on LTFU in this cohort (Kabarambi, Kansiime et al. 2022) found that individuals at higher risk of HIV—including young people (<25 years), females, recreational drug users, and those engaged in transactional sex—were more likely to be lost to follow-up. This suggests that our HIV incidence estimate may be an underestimate. This has now been acknowledged.

**Comment 7**. If this study is to plan for candidates for HIV vaccine study, then it would be nice to regress the predictors of those retained in care as these are possible participants with a high risk of HIV.

**Response**: We appreciate the reviewer’s comment. Our findings indicate that individuals lost to follow-up in this population were those at higher risk of HIV(Kabarambi, Kansiime et al. 2022).

**References:**

Ahmed, S., T. Lutalo, M. Wawer, D. Serwadda, N. K. Sewankambo, F. Nalugoda, F. Makumbi, F. Wabwire-Mangen, N. Kiwanuka and G. Kigozi (2001). "HIV incidence and sexually transmitted disease prevalence associated with condom use: a population study in Rakai, Uganda." Aids **15**(16): 2171-2179.

Armstrong, E. and R. Kaul (2021). "Beyond bacterial vaginosis: vaginal lactobacilli and HIV risk." Microbiome **9**(1): 239.

De Schacht, C., N. Mabunda, O. C. Ferreira Jr, N. Ismael, N. Calú, I. Santos, H. J. Hoffman, C. Alons, L. Guay and I. V. Jani (2014). "High HIV incidence in the postpartum period sustains vertical transmission in settings with generalized epidemics: a cohort study in Southern Mozambique." Journal of the International AIDS Society **17**(1): 18808.

Dellar, R. C., S. Dlamini and Q. A. Karim (2015). "Adolescent girls and young women: key populations for HIV epidemic control." Journal of the International AIDS Society **18**: 19408.

Donnell, D., S. Kansiime, D. V. Glidden, A. Luedtke, P. B. Gilbert, F. Gao and H. Janes (2023). "Study design approaches for future active-controlled HIV prevention trials." Statistical Communications in Infectious Diseases **15**(1): 20230002.

Gómez-Olivé, F. X., B. Houle, M. Rosenberg, C. Kabudula, S. Mojola, J. K. Rohr, S. Clark, N. Angotti, E. Schatz and K. Kahn (2020). "Brief report: HIV incidence among older adults in a rural South African setting: 2010–2015." JAIDS journal of acquired immune deficiency syndromes **85**(1): 18-22.

Joseph, S., P. Kaleebu, E. Ruzagira, C. H. Hansen, J. Seeley, V. Basajja, J. Weber, J. Fox, C. Kingsley, S. Aboud, E. Lyamuya, L. Maganga, D. Pamba, G. Ramjee, J. Yssel, E. Viegas, I. Jani, F. Priddy, C. Nilsson, A. Kroidl, G. Pantaleo, M. Robb, A. Crook, D. Dunne and S. Mccormack (2019). "OC 8491 PREPVACC: A PHASE III, MAMS ADAPTIVE PROPHYLACTIC HIV VACCINE TRIAL WITH A SECOND RANDOMISATION TO COMPARE F/TAF WITH TDF/FTC PREP." BMJ Global Health **4**(Suppl 3): A10-A10.

Kabarambi, A., S. Kansiime, S. Kusemererwa, J. Kitonsa, P. Kaleebu and E. Ruzagira (2022). "Predictors of loss to follow-up in an HIV vaccine preparedness study in Masaka, Uganda." International Journal of Environmental Research and Public Health **19**(11): 6377.

Kaleebu, P., P. K. Kitandwe, T. Lutalo, A. Kigozi, C. Watera, M. B. Nanteza, P. Hughes, J. Musinguzi, A. Opio and R. Downing (2018). "Evaluation of HIV-1 rapid tests and identification of alternative testing algorithms for use in Uganda." BMC Infectious Diseases **18**: 1-14.

Kasamba, I., J. Mugisha, A. Abaasa, R. Makanga, E. Ruzagira, P. Kaleebu, J. Seeley and R. Newton (2023). "Thirty years of change in HIV incidence among adults in the Kyamulibwa General Population Cohort in rural southwest Uganda, 1989-2021." International journal of infectious diseases **129**: 125-134.

Kharsany, A. B. M., C. Cawood, L. Lewis, N. Yende-Zuma, D. Khanyile, A. Puren, S. Madurai, C. Baxter, G. George, K. Govender, S. Beckett, N. Samsunder, C. Toledo, K. A. Ayalew, K. Diallo, M. Glenshaw, A. Herman-Roloff, E. Wilkinson, T. de Oliveira, S. S. Abdool Karim and Q. Abdool Karim (2019). "Trends in HIV Prevention, Treatment, and Incidence in a Hyperendemic Area of KwaZulu-Natal, South Africa." JAMA Netw Open **2**(11): e1914378.

Kusemererwa, S., S. Kansiime, G. Mutonyi, A. Namirembe, S. Katana, J. Kitonsa, A. Kakande, J. M. Okello, P. Kaleebu and E. Ruzagira (2021). "Predictors of oral pre-exposure prophylaxis (PrEP) uptake among individuals in a HIV vaccine preparedness cohort in Masaka, Uganda." Medicine **100**(44): e27719.

Kusemererwa, S., S. Kansiime, S. Nakamanya, E. Mbabazi, J. Fox, S. McCormack, P. Kaleebu and E. Ruzagira (2024). "Contraceptive use, prevalence and incidence of pregnancy and associated factors among women participating in a vaccine preparedness cohort study in Masaka, Uganda, a retrospective secondary analysis." Reproductive health **21**(1): 199.

Mlisana, K., N. Naicker, L. Werner, L. Roberts, F. Van Loggerenberg, C. Baxter, J.-A. S. Passmore, A. C. Grobler, A. W. Sturm and C. Williamson (2012). "Symptomatic vaginal discharge is a poor predictor of sexually transmitted infections and genital tract inflammation in high-risk women in South Africa." The Journal of infectious diseases **206**(1): 6-14.

Stannah, J., N. Soni, J. K. S. Lam, K. Giguère, K. M. Mitchell, N. Kronfli, J. Larmarange, R. Moh, M. Nouaman and G. M. Kouamé (2023). "Trends in HIV testing, the treatment cascade, and HIV incidence among men who have sex with men in Africa: a systematic review and meta-analysis." The lancet HIV **10**(8): e528-e542.

Uganda, M. o. H. (2022). CONSOLIDATED GUIDELINES FOR THE PREVENTION AND TREATMENT OF HIV AND AIDS IN UGANDA, dsduganda.

UNAIDS (2024). The urgency of now: AIDS at a crossroads. Geneva: Joint United Nations Programme on HIV/AIDS; 2024. . UNAIDS.

Westreich, D., N. Jamal, J. S. Smith, D. Schulze, S. Williams, P. Michelow, S. Levin and C. Firnhaber (2014). "Injectable and oral contraception and the incidence and progression of cervical disease in HIV-infected women in South Africa." Contraception **89**(4): 286-291.
