# Supplementary material for: Estimating HIV incidence and assessing associated risk factors among adults: Evidence from the 2018–2022 HIV vaccine preparedness cohort in Masaka, Uganda
Source: PLoS One. 2026 May 8;21(5):e0348769. doi: 10.1371/journal.pone.0348769 (PMC13155609; doi:10.1371/journal.pone.0348769)
Supplement: Attachment — Submitted filename: Response to Reviewers_30Jul2025.docx [file pone.0348769.s008.docx]

**Response sheet for submission ID PONE-D-24-44704 R2: Estimating HIV incidence and assessing associated risk factors among adults: Evidence from the 2018-2022 HIV vaccine preparedness cohort in Masaka, Uganda**

**Comments and responses**

We wish to immensely thank the reviewers for their valuable comments. Please find below a point-by-point response to the new set of comments raised.

**Reviewer #1**

Thank you again for the opportunity to review the revised version of this paper, which addresses a study with the potential to make a significant contribution to HIV research, particularly within the framework of the "First 95" initiative. I would like to thank the authors for the responses provided. Overall, most of my comments have been integrated, and the quality of the manuscript has improved accordingly. Nonetheless, some comments issues remain (particularly on methodological aspects) that need further attention from the authors to better improve the quality of the work. I also strongly recommend that in their response to reviewer comments, the authors systematically reference the specific line numbers of the revised manuscript. This would greatly facilitate the assessment of their revisions and allow a smoother review process. Below are my additional comments following the authors’ responses.

**Comment 1: Lines 154 – 160:** Thank you for these clarifications on my previous comment 5 and the additional details added in the manuscript regarding the screening algorithm and quality control of the HIV testing procedures. My concern was related in the fact that the national algorithm, although highly specific and sensitive, relies on rapid diagnostic tests that target antibodies, which may be present at low quantity during the early stages of infection (i.e., during seroconversion). Consequently, there remains a small probability that participants in the acute phase of infection could have been misclassified as HIV-negative at baseline and inadvertently included in the cohort. It would therefore be appropriate to acknowledge this as a potential limitation in the discussion section.

**Response:** We thank the reviewer for their insight. We have included this suggestion as a limitation in lines 358-361.

**Comment 2: Lines 180 – 182:** Thank you to the authors for their clarifications following my previous Comment 6. However, I respectfully disagree with the rationale provided, particularly the argument stating:

“The analytical approach used in this paper is a standard method for analyzing incidence data from a single study site. Similar approaches have been applied in other studies (Ahmed, Lutalo et al. 2001, De Schacht, Mabunda et al. 2014, Westreich, Jamal et al. 2014, Gómez-Olivé, Houle et al. 2020). If this were a multi-site study or one where the same individual was assessed multiple times for an outcome (e.g., repeated blood pressure measurements), incorporating random effects or using a GEE model would be appropriate. However, in our case, each participant had a single outcome (HIV status at the end of follow-up), and the study was conducted at a single site (Masaka). Therefore, there was no intra-individual variability or clear random effect to account for.”

This reasoning is not convincing for the following reasons:

1. The fact that the study was conducted at a single site does not in itself justify ignoring intra-individual variability. Even within a single site, a cohort of participants followed over time inevitably induces within-subject correlation across certain characteristics to each individual.

2. Similarly, the fact that each participant had a single binary outcome (HIV status) does not justify ignoring intra-individual variability, as several covariates (such as age, occupation, education level, marital status, and condom use at last sex (past 3 months)) may vary over time for a single participant and have been collected repeatedly throughout follow-up.

3. The statement, “If this were a multi-site study or one where the same individual was assessed multiple times for an outcome [...] incorporating random effects or using a GEE model would be appropriate”, appears to contradict the study design and reported results. According to the manuscript (line 104), participants were followed from 18 July 2018 to 31 December 2022. During this period, the same participants were followed over time, and several key characteristics (such as age, occupation, education level, marital status, residence, abnormal genital discharge in the past 3 months, and condom use) may have changed for a given participant over the four-year follow-up. This could be observed in the distribution of participant characteristics presented in Tables 1 and 2 (lines 266 and 289, respectively). Therefore, time-varying changes in these factors at the individual level could significantly influence HIV infection risk.

In light of the above, using a Poisson model with random effects or a marginal Poisson model using Generalized Estimating Equations (GEE) (with a random intercept and interaction terms with time for key variables) is both justified and methodologically appropriate for this study. Even if the results from this model was to remain similar to those currently presented, from a methodological standpoint, this approach better accounts for the longitudinal nature of the data and should be seriously considered by the authors.

**Response:** We thank the reviewer for these comments. We agree with your thoughts on this. The change in covariates (e.g demographics, risk behavior) over time can influence an individual’s risk of HIV infection over time. However, we are unsure as to whether the model you recommend is suitable for this analysis. It seems as though the suggested analysis would be suitable if there was intra-individual variability in the outcome of interest (HIV status in our case), not just intra-individual variability in the predictors (age, other demographics, risk behaviors etc.). Any publications investigating HIV incidence that used a similar approach to the one suggested may assist with this.

Alternatively, an analytical approach that would address your concerns would be incorporating time varying predictors in the survival analysis model used in our study. (Of note, we conducted survival analysis, assuming an exponential distribution - this is synonymous with “Poisson regression”). However, our study had a limited number of person years and events [For reference: Table 1 in the manuscript includes person years and HIV incident cases by the year of follow-up (First year, Second year and third year)]. Considering the limited data that was available beyond 1 year of follow-up, more detailed analyses investigating the impact of change in participant characteristics and behaviors over time on HIV incidence, are substantially underpowered/ limited. Additionally, participants had varying times of follow-up with some as little as 3 months, and others as long as 2 years or more, with only risk behaviour data collected every 6 months (other data were only collected at baseline). In consideration of the limited data, we opted to only conduct two main multi-variable analyses, one investigating the impact of baseline risk behaviors (adjusted for other predictors) and the other investigating the impact of the risk behavior data reported closest to the time-of sero-conversion or end of follow-up (also adjusted for other characteristics).

**Comment 3: Lines 154 – 160:** Thank you to the authors for the clarifications provided in response to my previous Comment 7 regarding the sensitivity analysis in light of the high loss to follow-up rate (22%). For the sensitivity analyses, the authors may formulate assumptions (ideally informed by a review of the literature) and present the corresponding results. A plausible assumption, in the lack of evidence to suggest otherwise, would be that the HIV incidence among participants lost to follow-up is similar to that observed among those who completed follow-up.

**Response:** We thank the reviewer for this comment. We acknowledged in the revised manuscript in lines 344-348 that “A major limitation of this study is that 22% of individuals did not attend any follow-up visits. These individuals were more likely to be young (<25years), female, recreational drug users, and persons who engage in transactional sex, subpopulations that are also associated with a higher risk of HIV acquisition. This likely introduced selection bias, potentially leading to an underestimation of HIV incidence in our study”.

**Comment 4: Lines 210 – 215:** Thank you for these clarifications following my previous Comment 8 and for the additional information provided in the footnote of Table 1. However, I am somewhat confused by the description: “§Other includes (multiple options allowed): Professional/technical worker (19%), sales/service worker (24%), subsistence agricultural worker (16%), craft and related trades worker (15%), house helper/labourer (16%), motorcyclist (8%), unemployed (7%), etc.; N/A, not applicable.” The sum of these percentages is 105%, and this is without considering the "etc." category. Could there be an error in these calculations? If so, please correct accordingly.

Additionally, reporting the HIV incidence within each of these subgroups would be valuable for readers.

**Response:** We thank the reviewer for this comment. The percentages add up to more than 100% because participants could list more than one occupation and be counted in more than one category. We have now added a supplementary table (S3 Tab) with HIV incidence in each of the different occupations.

**Comment 5: Lines 161 – 162:** Thank you to the authors for including the HIV diagnostic algorithm (S1 Fig). However, after reviewing it, I found the flow diagram somewhat confusing, particularly regarding the classification of positive and inconclusive cases. According to the algorithm, a participant is declared HIV-positive after reactive results on both the Determine and Stat-Pak tests. However, there is an arrow that subsequently leads to a "reactive" outcome followed by an "inconclusive" classification. This appears to be a misalignment or error in the linkage of results and arrows within the figure. I kindly ask the authors to review and correct this accordingly.

In addition, I would like to raise the following questions for clarification:

- Is the tie-breaker test (SD Bioline) routinely supplied by the Ministry of Health and available in all health facilities to ensure consistent implementation of this algorithm?
- As part of quality assurance procedures, is a second laboratory technician required to independently repeat the algorithm for a participant before an HIV-positive result is confirmed?

It would be helpful if the authors could clarify and include these details in the manuscript.

**Response:** We appreciate the reviewer’s observation regarding the figure misalignment and have corrected it accordingly.

We confirm that SD-Bioline serves as the designated tie-breaker test, as routinely provided by the Ministry of Health. It is readily available in health facilities across the country.

While the testing algorithm was not repeated by a second laboratory technician, this step is not required under the current national HIV testing algorithm. Nevertheless, to ensure quality, HIV testing procedures were performed by certified HIV testing service counsellors. Internal quality control (IQC) procedures were performed weekly using known positive and negative samples, while external quality assurance (EQA) assessments were conducted quarterly under the Uganda Virus Research Institute’s national reference laboratory EQA proficiency testing scheme (lines 164-168).

**Comment 6: Lines 210-218:** Thank you to the authors for including Supporting Table 2 in response to my previous Comment 27. As previously noted in table 1 (Line 266), the total percentage exceeds 100% for the “Other” occupation category: “¶Other includes (multiple options allowed): Professional/technical worker (19%), sales/service worker (24%), subsistence agricultural worker (16%), craft and related trades worker (15%), house helper/labourer (16%), motorcyclist (8%), unemployed (7%), etc.”

Please review and correct this accordingly.

**Response:** We thank the reviewer for their keen eye. The footnote in the supporting table 2 has been edited to remove the percentages as they didn’t belong to this table.

**Comment 7: Line 244 Fig 1:** Thank you for this clarification regarding my previous Comment 29, which reads as follows:

*“We have provided a detail to distinguish between the cohort and the trial in the methods section as written below: The main aim of the cohort study was to identify HIV-negative adults at high risk of HIV infection and prepare them for participation in a phase IIb HIV prophylactic vaccine trial (NCT04066881) at sites in Mozambique, South Africa, Tanzania, and Uganda. The trial aimed to determine the efficacy of two HIV prophylactic vaccine regimens and to compare the effectiveness of Truvada and Descovy as PrEP (Joseph, Kaleebu et al. 2019).”*

Does this imply that the 30 HIV-negative participants who completed the cohort study but were not enrolled in the PrEPVacc trial were no longer considered to be at high risk of HIV infection? If so, this seems somewhat surprising, given that being at high risk of HIV acquisition was an inclusion criterion for enrollment in the cohort study.

I recommend that the authors add a footnote to Figure 1 to clarify this subgroup (N = 30) and explain the reason for their non-enrollment in the PrEPVacc trial.

**Response:** The participants’ “risk of HIV infection” was re-evaluated at the time of screening for the trial, and they were considered to no longer be at elevated risk of infection as per their reported risk behaviours, or PrEP uptake status. A footnote has been added in Lines 240-242.

Notably, participation in a study that included regular HIV testing, quarterly counselling, and access to prevention information may have influenced participants’ sexual risk behaviours. Similar studies, including that involving this cohort, have reported declines in risky behaviour over time, likely due to consistent counselling (1, 2).

References

1. Kitonsa J, Kansiime S, Kusemererwa S, Onyango M, Nayiga B, Kabarambi A, Mugisha JO, Kaleebu P, Ruzagira E. Changes in self-reported risky sexual behaviour indicators among adults receiving regular risk reduction counselling and optional initiation of pre-exposure prophylaxis in an HIV vaccine preparedness study in Masaka, Uganda. Global Health Action. 2023 Dec 31;16(1):2242672.
2. Faini D, Hanson C, Baisley K, Kapiga S, Hayes R. Sexual behaviour, changes in sexual behaviour and associated factors among women at high risk of HIV participating in feasibility studies for prevention trials in Tanzania. PloS one. 2020 Apr 16;15(4):e0231766.
