# Supplementary material for: Estimating HIV incidence and assessing associated risk factors among adults: Evidence from the 2018–2022 HIV vaccine preparedness cohort in Masaka, Uganda
Source: PLoS One. 2026 May 8;21(5):e0348769. doi: 10.1371/journal.pone.0348769 (PMC13155609; doi:10.1371/journal.pone.0348769)
Supplement: Attachment — Submitted filename: Response sheet- Review3_Final19Mar2026.docx [file pone.0348769.s010.docx]

**Response sheet for Revision 3 of the manuscript entitled Estimating HIV incidence and assessing associated risk factors among adults: Evidence from the 2018-2022 HIV vaccine preparedness cohort in Masaka, Uganda.**

**Comment 1:** Thank you once again for the opportunity to review the second revised version of this manuscript, which addresses an important topic with strong potential to advance HIV research, particularly in the context of the “First 95” initiative. I appreciate the authors’ efforts in revising the paper and for the detailed responses provided. I acknowledge the authors’ efforts and the improvements made to the manuscript in response to earlier comments.

That said, despite progress, some key issues remain insufficiently addressed, most notably regarding methodological clarity and statistical rigor, as well as the presentation and consistency of the results in certain tables. These issues are critical and must be resolved to ensure the accuracy and scientific validity of the findings.

Below, I provide further comments and recommendations that require authors’ attention.

***Response:*** *We thank the reviewer for their interest in our manuscript and constructive comments. We appreciate the additional insights provided which have helped us improve the overall quality of the manuscript.*

**Comment 2: Lines 266–270:** Thank you for these additional details regarding my previous Comment 2, which concerns a fundamental methodological issue I have raised from the beginning of this manuscript revision process in my earlier review reports. I am therefore returning to it for the third time!

Regarding the authors’ response: “We agree with your thoughts on this. The change in covariates (e.g., demographics, risk behavior) over time can influence an individual’s risk of HIV infection over time. However, we are unsure as to whether the model you recommend is suitable for this analysis. It seems as though the suggested analysis would be suitable if there was intra-individual variability in the outcome of interest (HIV status in our case), not just intra-individual variability in the predictors (age, other demographics, risk behaviors etc.). Any publications investigating HIV incidence that used a similar approach to the one suggested may assist with this”:

- The authors should consider that in a random-effects Poisson model, it models the probability for a participant to have a given value of the outcome (here, HIV status: positive or negative). Thus, a participant might remain negative throughout the cohort follow-up, but their probability of being positive varies due to changes in other covariates for that same participant and other participants.
- There is ample evidence in the literature supporting this approach; a few references examples[1,2] are provided below

1	Laurent C, Yaya I, Cuer B, *et al.* Human Immunodeficiency Virus Seroconversion Among Men Who Have Sex With Men Who Use Event-Driven or Daily Oral Pre-Exposure Prophylaxis (CohMSM-PrEP): A Multi-Country Demonstration Study From West Africa. *Clin Infect Dis Off Publ Infect Dis Soc Am*. 2023;77:606–14. doi: 10.1093/cid/ciad221

2	M M, A B, Rm G, *et al.* Longitudinal population-level HIV epidemiologic and genomic surveillance highlights growing gender disparity of HIV transmission in Uganda. *Nat Microbiol*. 2024;9. doi: 10.1038/s41564-023-01530-8

Concerning the authors’ further arguments: “Alternatively, an analytical approach that would address your concerns would be incorporating time-varying predictors in the survival analysis model used in our study …… (also adjusted for other characteristics)” : this does not justify the a priori exclusion of using a Poisson model with random effects or a marginal Poisson model using Generalized Estimating Equations (GEE) which is the appropriate statistical approach for this study design, because the claimed lack of sufficient data / limited power beyond the first year of follow-up should be demonstrated by presenting estimates obtained from this model applied to the collected data, rather than simply being assumed.

Additionally, the statement: “*Of note, we conducted survival analysis, assuming an exponential distribution – this is synonymous with Poisson regression*” is misleading. While it is true that an exponential survival model (assuming a constant hazard) can produce estimates equivalent to a Poisson regression of events per person-time under certain conditions, the two approaches are not strictly synonymous. Poisson regression is fundamentally a model for count data (ideal for this study analysis), whereas the exponential survival model is a parametric time-to-event model. Equivalence only holds under the assumption of a constant hazard and when person-time is correctly incorporated as an offset in the Poisson model. Furthermore, this was not mentioned in the methodology section of the manuscript, which raises concern. So the authors should clearly clarify the statistical modelling approach actually used, and ensure it is accurately described in the methodology section of the paper.

***Response:*** *Thank you for these very insightful comments and the publications. We have now included a supplementary analysis (Supporting table 4) of the “Risk indicators association with HIV acquisition … (accounting for repeated assessments of risk at individual level over time)”. We utilised Mixed-effects Poisson Regression models with individual-specific random effects for that analysis.*

*Also, in the methodology section of the paper, we have now acknowledged that, “The natural log of the person-time (years) of follow-up was incorporated as an offset in the Poisson regression models”.*

**Comment 3: Lines 358–361:** Thank you to the authors for addressing my previous comment #1 in the revised R2 version of the manuscript. However, the clarification remains insufficient. The manuscript still does not clearly articulate the limitation related to the potential bias of inclusion participants already infected (in seroconversion) but misclassified as HIV-negative due to the reliance on the national antibody-based rapid testing algorithm. The authors should explicitly acknowledge this limitation of selection bias of participants and discuss its implications, given the fact that additional HIV tests were performed on participants quarterly after enrollment. This point could also be described in a separate paragraph, as it differs from the previously presented limitations in this section.

***Response:*** *We appreciate the reviewer for their suggestion. This limitation of using antibody-based rapid diagnostic tests has been clarified and rewritten as a separate paragraph which now reads as follows: HIV infection was diagnosed using antibody-based rapid diagnostic tests, which may not identify very recent infections, before seroconversion has occurred, potentially resulting in delayed diagnosis and misclassification of HIV status. Quarterly HIV testing substantially mitigated this risk by increasing the likelihood of detecting new HIV infections during follow-up, although some underestimation of HIV incidence may still have occurred. Lines 379-383*

**Comment 4: Lines 266–270:** Thank you to the authors for these clarifications regarding my previous Comment #4 and for adding Supporting Table 3 (S3 Table). However, the approach of allowing a participant to fall into multiple sub-categories within the “Other” occupation category and presenting the results as such raises concerns regarding interpretation for at least three reasons:

1. The total count for the “Other” category would then be at least 354 (i.e., 105% when summing the stated percentages without accounting for “etc.”), instead of 337. Consequently, the overall sample size would become at least 1,132 instead of 1,115. This artificially inflates the number of participants effectively enrolled and having completed at least one visit.
2. A single participant could contribute simultaneously to multiple sub-categories of the “Other” occupation category. This introduces ambiguity in interpreting incidence estimates. For example, if the participant identified as new HIV case (seroconverting) under the “House helper/labourer” category (S3 Table) is the same individual identified as new HIV case (seroconverting) under the “Sales/service worker” category because he selected the both category, this would inflate the incidence estimate for the “Other” occupation category.
3. This approach violates an underlying assumption of the statistical model used in the analysis, which stipulate that observations (participants) are independent and identically distributed (i.i.d).

In light of the above, I suggest that the authors should:

1. Classify each participant into a single occupation category (for example, by defining a rule such as selecting the first option chosen or the primary occupation recorded in the database) and specify the methodology used in the appropriate section of the paper;
2. Specify the number and percentage for each sub-category in S3 Tab within “Other” (e.g., Professional/technical worker: 66 (19%)) and ensure that the total count equals 337 and that percentages sum to 100%;
3. Adjust the participant counts and percentages for these sub-categories in the footnotes of Table 1 (line 268);
4. Refer the reader to S3 Table for additional details. In the current manuscript, no reference to S3 Table is made in the interpretation of Table 1 (lines 250–263).

***Response:*** *Thank you for these suggestions.*

*For the main analyses, including the regression models, participants were categorized into only four distinct occupation groups: (i) Sex workers (n=509), ii) Salon/bar/lodge workers (n=181), iii) Subsistence fisheries workers (88) and iv) Other occupations, (n=337) as shown in Table 1. The overall sample size was therefore 1,115 participants.*

*Because the number of participants in the ‘Other’ group category is large, Supplementary Table 2 and the footnotes of Table 1 provide further details of the occupations in this category. These comprise Professional/technical worker (64, 19%), sales/service worker (80, 24%), subsistence agricultural worker (55, 16%), craft and related trades worker (49, 15%), house helper/labourer (53, 16%), motorcyclist (26, 8%), unemployed individuas (24, 7%), and other occupations not listed (18, 15%). Participants were able to report more than one occupation; therefore, the total responses exceed 337, which is the total number of study participants in this category. Within the ‘Other’ group, each of the three HIV incident cases is unique and counted only in one occupational category; therefore, there is no risk of inflating the incidence estimate for any of these categories.*

*For the main analysis (Table 1), the other ‘Other’ occupational category was not further disaggregated because this would result in several small groups, making the analysis and interpretation of the results unnecessarily complex.*

*As noted above, the disaggregated groups are presented only in Supplementary Table 2 to provide additional detail on the ‘Other’ category. An explanation is also provided for why the number of responses exceeds the number of participants. Each of the three HIV infections is unique to its respective disaggregated occupational group. Moreover, these disaggregated groups were not included in the regression models; therefore, the assumption of independence is unlikely to be violated.*

*We have added a reference to Supplementary Table 2 in the footer of Table 1.*

**Comment 5: S2 Tab:** Thank you to the authors for addressing my previous Comment 6. However, the footnote reference “§ Formal/informal employment, family, spouse etc.” is ambiguous, as the symbol “§” does not appear anywhere in the S2 Tab. The authors should insert the symbol in the appropriate location within the table before referencing it in the footnote, or remove the footnote if it is not necessary.

***Response:*** *-	Thank you for this observation. The symbol has been removed from the updated Table (now Supplementary Table 1).*

**Comment 6: Lines 266-270 and 289-292:**

1. The footnote in Tables 1 and 2 (“n, number of HIV infections;”) is ambiguous, as both tables contain two columns labeled “n (%)” and “n.” However, these two columns do not refer to the same information. I recommend that the authors correct this to avoid confusion.

***Response:*** *Thank you for this comment. We have revised Tables 1 and 2 to indicate that the second column shows the number and percentage of participants in each category, while the third column shows the number of HIV infections.*

2. In addition, the authors should provide clarification on the points below:
   a. The “n (%)” total is identical in both tables (1115 (100%)), although Table 1 presents baseline characteristics and Table 2 presents characteristics at the end of cohort follow-up. Given that the authors reported a 22% loss to follow-up in the cohort, one would expect the sample size to decrease in Table 2. What explains this?

***Response:*** *Thank you for this comment. The* n (%)” total is identical in both tables (1115) because this is the number of participants who had follow-up data i.e., attended at least one follow up visit. *Table 1 presents the associations between HIV incidence and baseline risk indicators, while Table 2 presents the associations between HIV incidence and the most recent HIV risk data collected from each participant during follow-up. The 22% (n=307) refers to the participants who did not attend any follow-up visits post-enrolment and is based on the total of 1422 individuals enrolled, as reported elsewhere in the manuscript (Baseline characteristics; Figure 1). Detailed baseline characteristics for all enrollees are provided in Supplementary Table 1*

b. The variable “Gender,” which was statistically significant in Table 1, does not appear in Table 2. However, the lines 194-197 clearly state that “Age and gender were included in the multivariable models a priori based on established evidence from previous research, which consistently identifies them as major key risk factors for incident HIV [26-28].” Why, then, is gender absent from Table 2?

***Response:*** *Thank you for this comment. Table 2 was intended to present the associations between HIV incidence and risk indicator data collected at the final follow-up assessment. Age, gender, residence, and occupation were included a priori as adjustment variables in the multivariable models. For presentation purposes, these variables were initially not included in Table 2 because their associations with HIV incidence were already shown in Table 1. However, we agree that presenting the adjusted estimates in these models facilitates comparsion between baseline and follow-up analyses. We have therefore now included the results for these variables in Table 2 and in Supporting 4_Table (analysis accounting for repeated assessments).*

c. Each variable in the “n (%)” column of Tables 1 and 2 sums to 1115 (100%), which suggests that there were no missing values (a scenario highly unlikely in cohort studies) or that missing values were imputed. If imputation was performed, the authors should specify the imputation methods used in the methodology section (statistical analysis). Could the authors clarify this!

***Response:*** *Thank you for your comment. No imputation was performed for this analysis. The study database underwent routine data cleaning and automated data quality checks throughout data collection. Any missing or inconsistent entries generated queries that were sent to the research teams for resolution using source documents as close to real time as possible. As a result of this process, all variables included in the analyses presented in Tables 1 and 2 had complete data. If missing data had occurred, it would have been acknowledged in the footnotes of each table.*

*We have now clarified our data management process in the methods section. See lines 158-164.*
