# Supplementary material for: Estimating HIV incidence and assessing associated risk factors among adults: Evidence from the 2018–2022 HIV vaccine preparedness cohort in Masaka, Uganda
Source: PLoS One. 2026 May 8;21(5):e0348769. doi: 10.1371/journal.pone.0348769 (PMC13155609; doi:10.1371/journal.pone.0348769)
Supplement: Attachment — Submitted filename: Plos1 Review comments150426.odt [file pone.0348769.s011.odt]

Plos1 Review comments
Reviewer: DR. Joseph Mugisha Okello
Title: estimating HIV incidence and assessing associated risk factors among adults:
Evidence from the 2018-2022 HIV vaccine preparedness cohort in Masaka, Uganda
Thank you very much for considering me to review the manuscript. I have looked at all the comments made by the previous reviewer and I am happy that the authors have addressed all of the comments fully. I only have one major concern This study was conducted between 2018 and 2022.  This was almost 5 years ago.  Given that there are several HIV prevention technologies that have been used since then, including PrEP, do you think the HIV incidence that was estimated using these data is still valid currently?  

Minor: Although the authors acknowledge the limitation of around 22% of the enrolled participants not attending at least one follow up visit, it’s not clear how a low number (38%) that completed the study could have affected the outcomes.

Joseph Mugisha (PhD)
